# Salvage Stereotactic Radiosurgery for Multiple Brain Recurrences: How Much is Enough?

**DOI:** 10.7759/cureus.1835

**Published:** 2017-11-10

**Authors:** Fred Hsu

**Affiliations:** 1 Radiation Oncology, BC Cancer Agency – Abbotsford Centre

**Keywords:** stereotactic radiosurgery, salvage, brain metastases

## Abstract

Stereotactic radiosurgery (SRS) can be used as a salvage treatment in selected patients with recurrent brain metastases after previous brain radiation. We report the case of a patient with metastatic lung adenocarcinoma who experienced recurrence numerous times in the brain and was successfully treated each time with SRS. For this patient, brain imaging surveillance helped identify metastases early for salvage SRS. We have also included a discussion of published literature regarding the neurocognitive toxicity of repeated courses of SRS.

## Introduction

Radiotherapy is the primary treatment for intracranial metastases. For recurrent brain disease following initial whole brain radiotherapy (WBRT) or stereotactic radiosurgery (SRS), some patients can be treated with another course of SRS. The safety and efficacy of repeated courses of SRS is not well known. Here we report an exceptional outcome of a patient initially treated with WBRT and multiple brain recurrences treated with multiple courses of SRS.

## Case presentation

**Figure 1:**
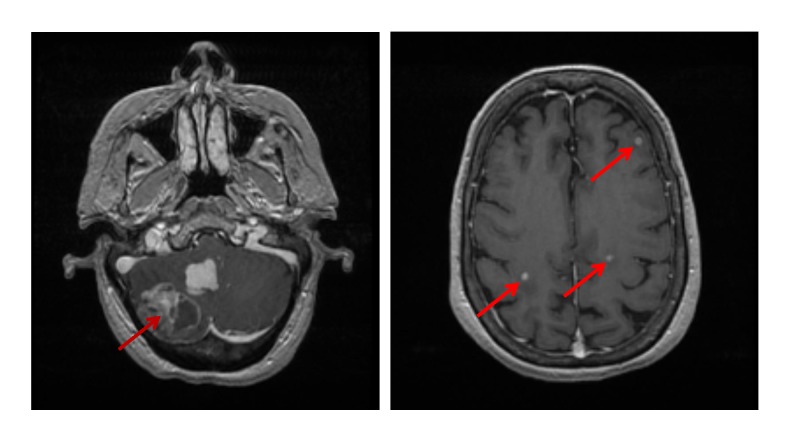
MRI scans of the brain of the patient at initial presentation Brain metastases are highlighted by arrows. Two large metastases are seen in the cerebellum (left image). Additional small metastases are diffusely distributed (right image). The patient subsequently had whole brain radiation.

A 60-year-old female, South-Asian never-smoker presented in December 2011 with dizziness and nausea. A magnetic resonance imaging (MRI) scan of the head detected 25 brain metastases. The two largest metastases were in the right cerebellum measuring 38 mm and 22 mm in size; the remaining were < 10 mm with a diffuse bilateral cerebral and cerebellar distribution. The patient’s initial MRI is presented in Figure 1. Neurosurgical resection of the largest cerebellar metastasis showed poorly differentiated adenocarcinoma pathology, CK7 and TTF-1 positive, consistent with a lung primary. Genetic analysis did not detect the presence of an epidermal growth factor receptor (EGFR) exon 21 or exon 19 or ALK mutation. The primary tumour was identified in the left lower lobe lung and there were no other extracranial metastases on computed tomography (CT) imaging of the body.

**Figure 2:**
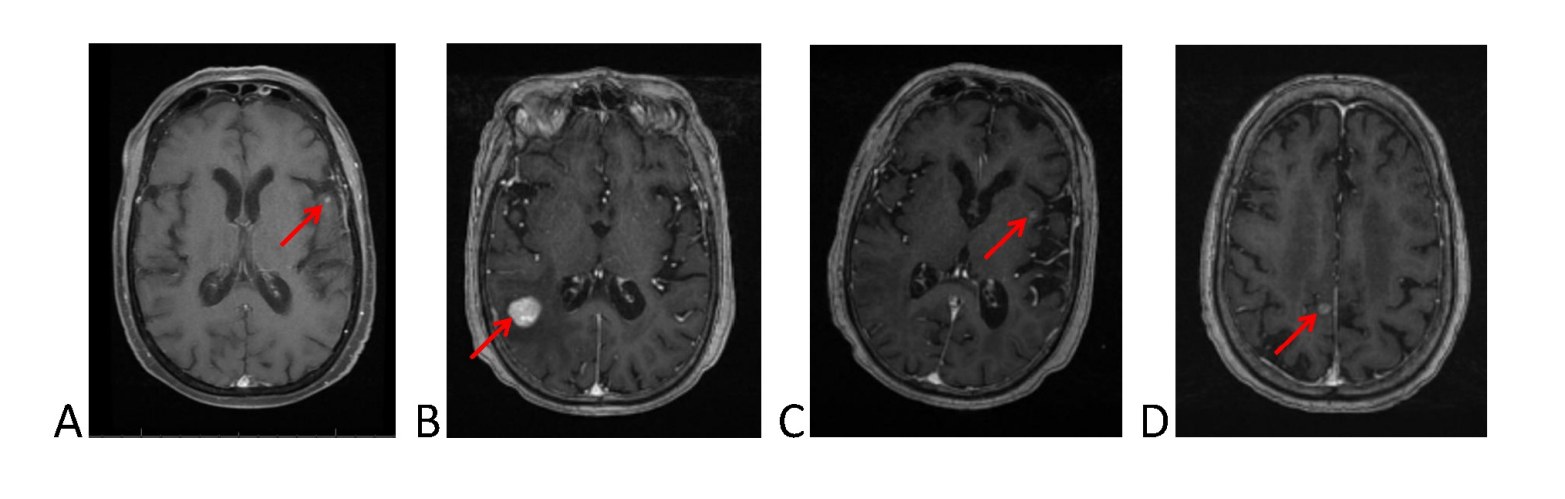
MRI scans of the brain of the patient at nine, 30, 34, and 44 months (images A, B, C, D, respectively) New brain metastases are highlighted by arrows. The patient had stereotactic radiosurgery for each new brain metastasis.

The patient was treated with WBRT (20 Gy in five fractions). A CT scan of the head two months after WBRT showed an empty resection cavity, and definite intracranial lesions were no longer visible. The patient received platinum-based chemotherapy for four months with regression of the primary lung tumour. The patient did not receive further maintenance systemic therapy and was followed with chest and brain imaging every three to six months using both CT and MRI. At nine months, an MRI showed new asymptomatic brain metastases in the left frontal lobe (3 mm in size) and the left parietal lobe (8 mm) (Figure 2A). The two lesions were treated with SRS to a dose of 24 Gy in a single fraction (24 Gy/1). At 30 months, she developed a new metastasis in the right parietal lobe (16 mm) (Figure 2B). This was treated with SRS to a dose of 24 Gy/1. At 34 months, an MRI showed a new lesion in the left temporal lobe (5 mm) (Figure 2C), which was treated with SRS (24 Gy/1). At 44 months, brain imaging showed a fourth recurrence at new sites in the right parietal lobe (6 mm) (Figure 2D) and left temporal lobe (3 mm). The first lesion was treated with 24 Gy/1; the second lesion was treated with 18 Gy/1 because of the proximity to a lesion previously treated with SRS. The patient died at 56 months from the time of her initial pathologic diagnosis. At the time of her death, there was radiographic progression of the right parietal lobe lesion treated 26 months prior, while all other treated brain lesions were no longer visible. There was no recurrence of disease in the thorax. The only site of extracranial progression was lymphadenopathy in the neck in which there was a complete clinical response to palliative radiotherapy.

The SRS technique at our institution uses a frameless stereotactic mask system. Patients have a contrast enhanced CT and MRI scan to delineate the gross tumour volume and a 1.5 mm isotropic expansion for the planning target volume (PTV). Planning is performed on the BrainScan/iPlan stereotactic planning software (Brainlab AG, Germany) using multiple arcs and 3 mm multileaf collimation. The dose prescription is to the 80% isodose volume covering the PTV.

Clinically, the patient was asymptomatic and back to her normal baseline functional status by six weeks after WBRT. At 23 months, she reported bilateral hearing loss. Audiology showed profound bilateral sensorineural hearing loss in the 1000 Hz range. At 28 months, her family reported a decline in short-term memory. This was followed by a gradual progression of forgetfulness, anxiety, personality changes, and a lack of initiative, which did not coincide with the presence or absence of brain metastases. At the end of her life she suffered from a severe dementia. The specific cause of death was not known and an autopsy was not performed.

## Discussion

We present the case of a patient with multiple brain recurrences who had exceptional intracranial disease responses to WBRT and four separate courses of SRS over the span of four years. In total, the patient had 31 brain metastases treated. We attribute her lengthy clinical course to a favorable extracranial disease behaviour and good intracranial control with radiotherapy. The patient’s female gender, Asian ethnicity, and never-smoking status support the suggestion that she was a carrier of a genetic mutation. Pathology from the patient’s brain metastasis did not detect mutations in EGFR exon 19 or exon 21 or anaplastic lymphoma kinase (ALK), which comprise the majority of known driver mutations in lung adenocarcinoma. One explanation is a recognized heterogeneity in EGFR mutation status between the pulmonary primary and the brain metastases [1]. Alternatively, the patient may have harboured a less common driver mutation (but unknown) to explain the favourable disease biology. Intracranial disease control with salvage radiotherapy may be of particular importance in patients with driver mutations that confer a better systemic prognosis.

Stereotactic radiosurgery as salvage treatment for intracranial recurrences is becoming increasingly important for several reasons. Patients are living longer with extracranial disease because of therapeutic advances in systemic therapy. Stereotactic radiosurgery without WBRT has gained traction as the mainstay of upfront management [2], but the omission of WBRT is associated with a higher rate of intracranial recurrence [3]. Identifying which patients might benefit most from salvage SRS, as opposed to salvage WBRT, will need further study. Implications for follow-up monitoring will also be important. This patient maintained brain imaging surveillance during her clinical course and most brain recurrences were identified while small in size and few in number such that they were suitable for SRS. A study reported by Shen, et al. [4] of patients treated with salvage SRS also found that new brain lesions infrequently caused neurologic symptoms before routine imaging detection, and detecting brain lesions early may have advantages.

The patient developed dementia by the end of her life, which is a concern for repeated brain radiation. Although we suspect the patient’s cognitive deterioration was predominantly the result of the initial WBRT, the impact of multiple courses of SRS is uncertain. The risk of neurocognitive impairment associated with WBRT is well recognized in literature [5-6]. To our knowledge, there is no published data regarding the risk of neurocognitive impairment with repeated courses of SRS for multiple brain recurrences. However, the current literature suggests that SRS for multiple brain lesions is safe. In a study examining long-term cognitive function in patients with 1-10 brain metastases treated with SRS, the maintenance of mini-mental state examination scores did not appear to differ with the number of brain metastases treated [7]. In this study, leukoencephalopathy was detected primarily in patients after salvage WBRT. In a study of patients treated with two or more courses of SRS, Shultz, et al. [8] reported grade 2-4 adverse radiation events in only 2% of lesions. This study, however, did not include formal neurocognitive and quality of life assessments. Disease location in eloquent brain areas and tumour size may also be important factors. In a treatment planning study with Gamma Knife radiosurgery, Hatiboglu, et al. [9] found that the total tumour volume was a more significant predictor of mean whole brain dose and whole brain integral dose compared to the number of lesions.

## Conclusions

Stereotactic radiosurgery can be a very effective salvage treatment for recurrent brain metastases if detected while small in size and few in number. The neurocognitive impact of repeated courses of SRS is uncertain and needs further study.
